# Left ventricular long axis function assessed during cine-cardiovascular magnetic resonance is an independent predictor of adverse cardiac events

**DOI:** 10.1186/s12968-016-0257-y

**Published:** 2016-06-07

**Authors:** Vibhav Rangarajan, Satish Jacob Chacko, Simone Romano, Jennifer Jue, Nikhil Jariwala, Jaehoon Chung, Afshin Farzaneh-Far

**Affiliations:** Department of Medicine, Section of Cardiology, University of Illinois at Chicago, 840 South Wood St. M/C 715, Suite 920S, Chicago, IL 60612 USA; Department of Medicine, University of Verona, Verona, Italy; Department of Medicine, Division of Cardiology, Duke University, Durham, NC USA

**Keywords:** Cardiovascular magnetic resonance, Prognosis, Left ventricular function, Longitudinal strain, Mitral annular plane systolic excursion, Atrioventricular plane displacement (AVPD)

## Abstract

**Background:**

Left ventricular pump function requires a complex interplay involving myocardial fibers orientated in the longitudinal, oblique and circumferential directions. Long axis dysfunction appears to be an early marker for a number of pathological states. We hypothesized that mitral annular plane systolic excursion (MAPSE) measured during cine-cardiovascular magnetic resonance (CMR) reflects changes in long axis function and may be an early marker for adverse cardiovascular outcomes. The aims of this study were therefore: 1) To assess the feasibility and reproducibility of MAPSE measurements during routine cine-CMR; and 2) To assess whether MAPSE, as a surrogate for long axis function, is a predictor of major adverse cardiovascular events (MACE).

**Methods:**

Four hundred consecutive patients undergoing CMR were prospectively enrolled. MAPSE was measured in the 4-chamber cine view. Patients were prospectively followed for major adverse cardiac events (MACE) - death, non-fatal myocardial infarction, hospitalization for heart failure or unstable angina, and late revascularization. Cox proportional hazards regression modeling was used to identify factors independently associated with MACE. Net reclassification improvement (NRI) was calculated to assess whether addition of MAPSE resulted in improved risk reclassification of MACE.

**Results:**

Seventy-two MACE occurred during a median follow-up of 14.5 months. By Kaplan-Meier analysis, patients with lateral MAPSE <1.11 cm (median) experienced significantly higher incidence of MACE than patients with a MAPSE ≥1.11 cm (*p* = 0.027). After adjustment for established clinical risk factors which were univariate predictors (age, diabetes, hypertension, NYHA class, LV mass), lateral MAPSE remained a significant independent predictor of MACE (HR = 4.384 per cm decrease or 1.344 per 2 mm decrease; *p* = 0.020). Incorporation of lateral MAPSE into this risk model resulted in a net reclassification improvement (NRI) of 0.18 (*p* = 0.006).

**Conclusions:**

Reduced long axis function assessed with lateral MAPSE during cine-CMR is an independent predictor of MACE.

## Background

Left ventricular pump function requires a complex interplay involving myocardial fibers orientated in the longitudinal, oblique and circumferential directions [1–4]. This results in a combination of circumferential shortening, radial wall thickening, and long axis shortening. Long axis atrio-ventricular plane displacement has been shown to be a major contributor to left ventricular pumping in both health and disease [5, 6]. In addition, through displacement of the atrio-ventricular annulus, long axis function seems to affect myocardial function in unique ways - including mediating left ventricular diastolic filling, and in expediting atrial filling from the great veins [6, 7]. Long axis dysfunction appears to be an early marker for a number of pathological states [8]. Moreover, assessment of longitudinal function using echocardiography has been shown to provide independent prognostic information in a wide variety of cardiac conditions [9, 10]. Despite these important and unique features, long axis function is not routinely assessed or reported during clinical Cardiovascular Magnetic Resonance (CMR) [11].

We hypothesized that mitral annular plane systolic excursion (MAPSE) measured during cine-CMR imaging reflects changes in long axis function and may be an early marker for adverse cardiovascular outcomes. The aims of this study were therefore: 1) To assess the feasibility and reproducibility of MAPSE measurements during routine cine-CMR; and 2) To assess whether MAPSE, as a surrogate for long axis function, is a predictor of major adverse cardiovascular events (MACE).

## Methods

### Study population

Four hundred consecutive outpatients referred for clinical CMR were prospectively enrolled at a single academic medical center. Patients were excluded if they had metallic implants incompatible with CMR, glomerular filtration rate < 30 ml/min, severe claustrophobia, or mitral valve replacement. Information on baseline demographic variables and prior laboratory testing was obtained from patient interviews and the electronic medical record. Patients gave informed written consent for the protocol, which was approved by the local institutional review board.

### CMR acquisition

Images were acquired on a 3 T scanner (Philips Achieva, Philips Medical Systems, Best, the Netherlands) using a six-element phased-array receiver coil. Steady-state free-precession cine images were acquired in multiple short-axis and three long-axis views (repetition time, 3.0 ms; echo time, 1.5 ms; flip angle, 40°; slice thickness 6 mm). Short-axis views were obtained every 1 cm to cover the entire left ventricle. Gadolinium contrast (0.15 mmol/kg gadoteridol, Bracco Diagnostics) was administered and delayed enhancement CMR (DE-CMR) was performed 10-15 min later with a 2D segmented gradient echo phase-sensitive inversion-recovery sequence in the same views used for cine-CMR. Inversion delay times were typically 280 to 360 ms

### CMR analysis and MAPSE assessment

**Fig. 1:**
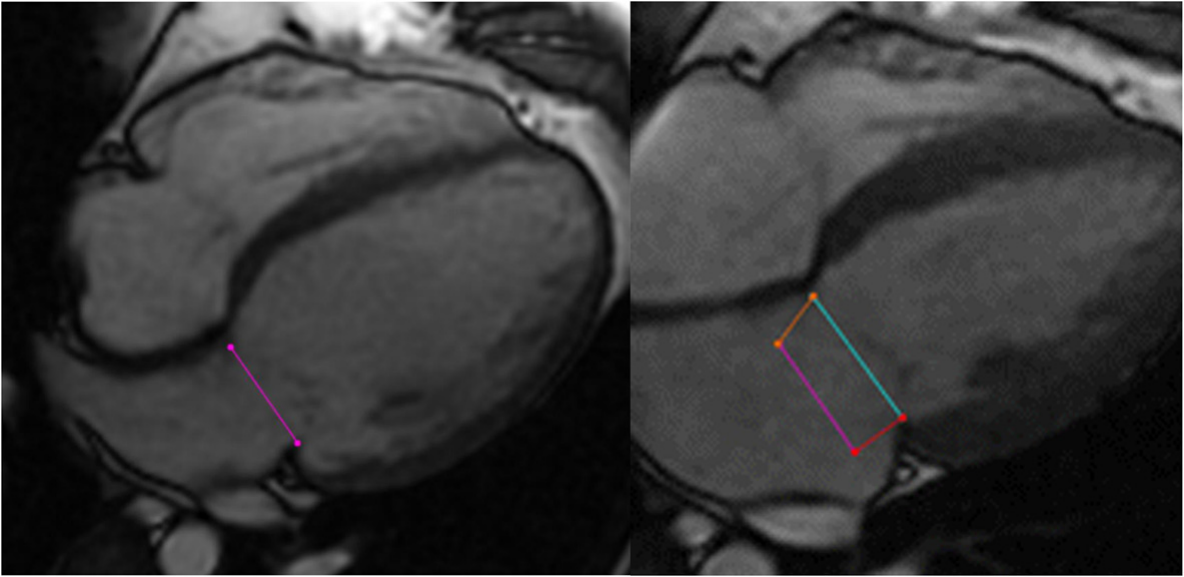
Measurement of MAPSE. Septal and lateral mitral annular positions were recorded at end diastole (left panel*, purple line*) and end systole (right panel*, blue line*), allowing for assessment of lateral (right panel, *red line*) and septal MAPSE (right panel, *orange line*)

Septal and lateral mitral annular positions were marked at end-diastole in the 4-chamber view (Fig. 1). The images were advanced frame-by-frame to end-systole (just before mitral valve opening) where septal and lateral mitral annular positions were again identified. Septal MAPSE was defined as the distance between the septal mitral annular position at end-diastole to the septal mitral annular position at end-systole. Lateral MAPSE was defined as the distance between the lateral mitral annular position at end-diastole to the lateral mitral annular position at end-systole (Fig. 1). Thus MAPSE was measured as the simple linear displacement between end-diastole and end-systole. Measurements were manually performed by a CMR physician who was blinded to patient information and outcomes. In 50 randomly selected patients, a second blinded CMR physician measured MAPSE for assessment of inter-observer variability. In another 50 randomly selected patients, the same physician re-measured MAPSE in a blinded fashion for assessment of intra-observer variability.

The presence and location of LGE was determined by visual inspection using the AHA 17-segment model. Regional enhancement was scored according to the visual spatial extent of enhanced tissue within each segment (0  =  no enhancement; 1  =  1–25 % enhanced; 2  =  26–50 %; 3  =  51–75 %; and 4  =  76–100 %) as described previously [12–16]. The analyses were performed using WebPAX (Heart IT, Durham, North Carolina).

### Follow-up

Patients were followed for the combined primary outcome of major adverse cardiac events (MACE) - death, non-fatal myocardial infarction, hospitalization for heart failure or unstable angina, and late revascularization (>90 days after CMR). Two cardiologists blinded to CMR results performed all standardized follow-up procedures. Clinical follow-up was obtained by review of the electronic medical records. In cases where records were not found in the medical chart, treating physicians and patients were contacted using a standardized questionnaire. Non-fatal myocardial infarction was defined by the presentation of an acute coronary syndrome and elevation of cardiac biomarkers (>99th percentile of the upper limit of normal), temporally consistent with an acute injury. The definition of heart failure hospitalization required the presence of an elevated B-natriuretic peptide (BNP) level in addition to signs and symptoms of heart failure. Unstable angina was defined by hospitalization for chest pain plus either coronary angiography revealing stenosis of >70 % or ischemia on noninvasive stress imaging performed during the hospitalization. The Social Security Death Index was used to confirm all cases of death. Time to event was calculated as the period between the CMR study and the first occurrence of a MACE. Patients who did not experience MACE were censored at time of last follow-up.

### Statistical analysis

Normally distributed data were expressed as mean ± SD. Continuous variables were compared by the Student’s *t*-test or Wilcoxon rank-sum (depending on data normality). Comparisons of discrete variables were made using the chi-square test; Fisher’s exact test was used when the assumptions of the chi-square test were not met. Inter and intra observer variability was analyzed using the Bland-Altman method [17]. Kaplan-Meier methods were used to evaluate time to the primary outcome of MACE. Cox proportional hazards regression modeling was used to identify factors that were independently associated with MACE. For the multivariable model, established clinical risk factors which were univariate predictors (at *p* ≤ 0.10) were considered as candidate variables. To assess the added prognostic value of lateral MAPSE, the final model was compared with a model in which lateral MAPSE was not included. The global chi-square statistic was calculated for both models, and compared using the likelihood ratio test. Model discrimination was compared by calculating the integrated discrimination improvement (IDI), which measures the improvement in sensitivity and specificity of the model with addition of the new predictor (lateral MAPSE) [18, 19]. Formal risk reclassification analyses were conducted by examining net reclassification improvement (NRI) using categories of <10 %, 10–15 %, and >15 %/year MACE rates to define low, intermediate, and high-risk categories, respectively [18]. A *p* value of <0.05 was considered statistically significant.

## Results

### Patient characteristics

Table 1 summarizes baseline patient characteristics. The mean age of the study population was 58 ± 15 years. Forty-five percent of patients were male and 33 % had diabetes mellitus. Thirty-one percent had known coronary artery disease, and 18 % were current smokers. Thirty-one percent had a history of heart failure and the mean ejection fraction was 59 ± 14 %.

**Table 1:** Baseline Characteristics Stratified by Median Level of MAPSE

Characteristics	Total	Lateral MAPSE < median	Lateral MAPSE ≥ median	*P* Value
	*N* = 400	*N* = 195	*N* = 205	
Age (±SD)	57.9 (±14.7)	59.7 (±14.6)	56.2 (±14.6)	0.017
Male %	44.8	47.7	42.0	0.248
BMI (±SD)	30.6 (±6.4)	31.0 (±6.5)	31.1 (±6.3)	0.876
Diabetes %	32.5	38.5	26.8	0.013
Hyperlipidemia %	52.0	56.9	47.3	0.055
Smoking %	18.0	23.6	12.7	0.005
Hypertension %	73.8	76.4	71.2	0.238
Known CAD %	31.1	36.6	25.9	0.020
Prior MI %	13.3	16.4	10.2	0.069
Prior PCI %	15.8	19.5	12.2	0.045
Prior CABG %	3.8	3.1	4.4	0.490
NYHA class	0.6 (±0.9)	0.5 (±0.9)	0.6 (±1.0)	0.295
Heart Failure %				
None	69.2	70.7	67.8	0.543
NYHA 1	12.1	12.6	11.6	0.760
NYHA 2	12.3	11.5	13.1	0.642
NYHA 3	5.4	4.7	6.0	0.564
NYHA 4	0.5	0.5	0.5	0.977
Antiplatelet Drug %	53.3	52.5	54.1	0.788
Statin %	50.1	49.6	52.1	0.684
ACE inhibitor %	40.4	43.9	40.0	0.235
Beta Blocker %	64.2	64.7	63.7	0.853
Diuretic %	46.5	42.0	50.7	0.144
LVEF (±SD)	58.9 (±13.7)	55.5 (±15.6)	62.2 (±10.7)	<0.001
LV mass g (±SD)	118.8 (±43.3)	123.6 (±48.1)	114.6 (±38.3)	0.039
LGE present %	20.9	26.7	15.3	0.006

*ACE* angiotensin converting enzyme, *BMI* body mass index, *CABG* coronary artery bypass grafting, *CAD* coronary artery disease, *LGE* late gadolinium enhancement, *LVEF* left ventricular ejection fraction, *MAPSE* Mitral Annular Plane Systolic Excursion, *MI* myocardial infarction, *NYHA class* New York Heart Association Class (class 0 signifies no heart failure), *PCI* percutaneous coronary intervention, *SD* standard deviation

Median MAPSE for the population was 1.11 cm. There was a significantly higher percentage of smokers, diabetics and patients with prior MI or known CAD in those individuals with MAPSE < median. Moreover, LVEF was significantly higher in patients with MAPSE ≥ median.

### Inter and intra observer variability

**Fig. 2:**
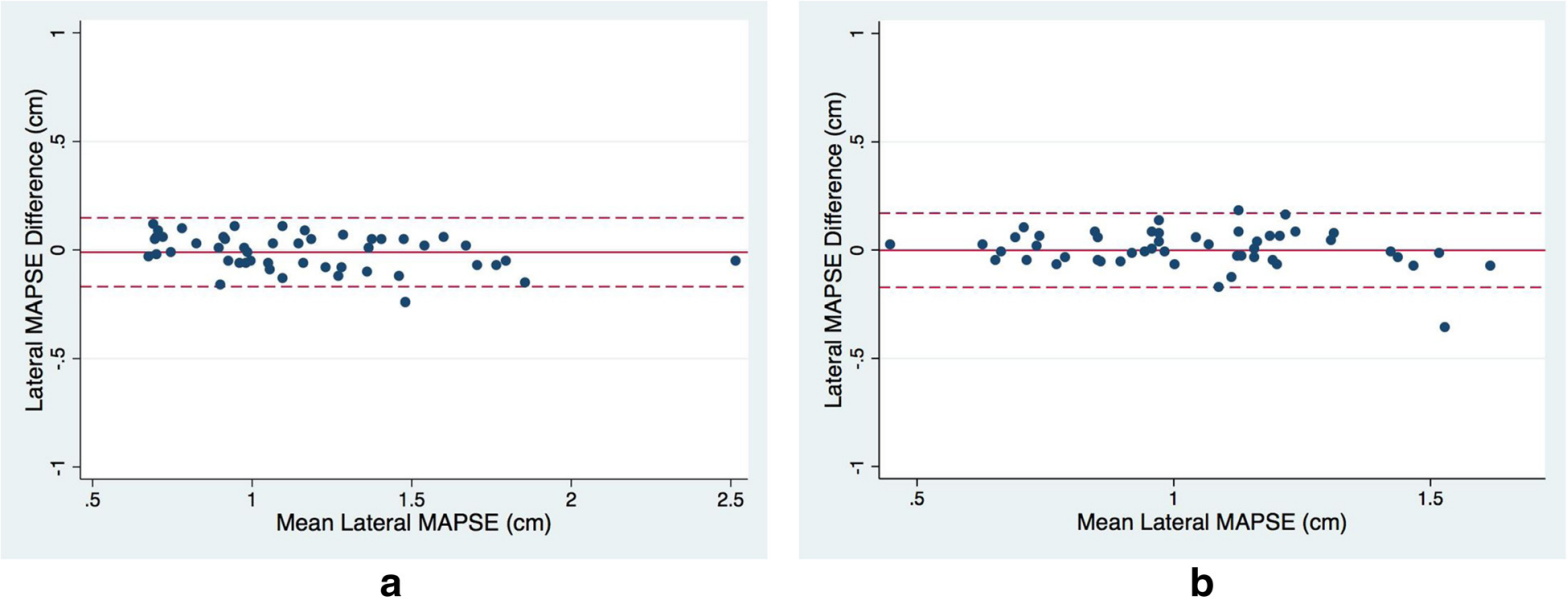
Bland-Altman analysis of lateral MAPSE for **a** interobserver and **b** intraobserver variability. Solid line represents the bias. Dashed line represents the limits of agreement

Bland-Altman analysis of interobserver repeatability for lateral MAPSE showed a bias of −0.01 cm. Limits of agreement were −0.17 to 0.15 cm. The Bland-Altman plot showed no systematic bias (Fig. 2a). Bland-Altman analysis of intraobserver repeatability for lateral MAPSE showed a bias of −0.002 cm. Limits of agreement were −0.17 to 0.17 cm. The Bland-Altman plot showed no systematic bias (Fig. 2b).

### Primary outcomes

A total of 72 major adverse cardiac events (MACE) occurred during a median follow-up of 14.5 months. This was comprised of 11 deaths, 10 non-fatal myocardial infarctions, 12 hospitalizations for heart failure, 28 hospitalizations for unstable angina, and 11 late revascularizations.

### Outcomes stratified by MAPSE

**Fig. 3:**
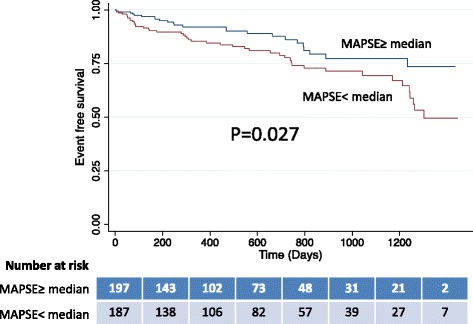
Kaplan-Meier curves for MACE, in patients with lateral MAPSE above and below the median. The number of patients at risk at each time interval for each group is presented

**Fig. 4:**
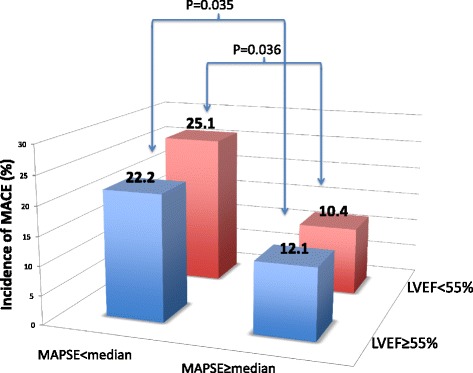
Overall incidence of MACE stratified by lateral MAPSE and LVEF

Overall, patients with lateral MAPSE < median had twice the number of cumulative MACE when compared to those with lateral MAPSE ≥ median (24 % vs 12 %, *p* = 0.0018). By Kaplan-Meier analysis, patients with lateral MAPSE < median experienced significantly higher incidence of MACE than patients with lateral MAPSE ≥ median (log-rank *p* = 0.027) (Fig. 3). Figure 4 shows the cumulative incidence of MACE stratified by MAPSE and LVEF. MACE occurred significantly more frequently in individuals with MAPSE < median, irrespective of LVEF. The highest cumulative incidence of MACE occurred in those with MAPSE < median and LVEF < 55 %. By univariable analysis, age, diabetes, hypertension, NYHA class, LV mass and lateral MAPSE were associated with occurrence of MACE (at *p* ≤ 0.10) (Table 2).

**Table 2:** Univariable and multivariable predictors of MACE

Variables	Univariable		Multivariable	
	Hazard Ratio (95 % CI)	*P* Value	Hazard Ratio (95 % CI)	*P* Value
Age	1.016 (0.999–1.033)	0.0585	0.985 (0.958–1.013)	0.286
Male	1.014 (0.638–1.612)	0.9531	-	-
Diabetes	1.693 (1.058–2.713)	0.0309	0.863 (0.428–1.740)	0.680
Hyperlipidemia	1.326 (0.825–2.131)	0.2397	-	-
Smoking	0.998 (0.555–1.795)	0.9953	-	-
Hypertension	2.482 (1.189–5.181)	0.0066	2.355 (0.820–6.766)	0.112
NYHA class	0.810 (0.618–1.060)	0.1080	0.853 (0.519–1.403)	0.532
LVEF	1.001 (0.985–1.017)	0.9234	-	-
LGE present	1.263 (0.757–2.110)	0.3794	-	-
LV mass	1.009 (1.002–1.017)	0.0211	1.008 (1.000–1.015)	0.044
Lateral MAPSE^a^	2.228 (1.051–4.679)	0.0331	4.384 (1.257–15.271)	0.020
Septal MAPSE^a^	1.585 (0.682–3.715)	0.2865	-	-

*LGE* late gadolinium enhancement, *LV* left ventricular, *LVEF* left ventricular ejection fraction, *MAPSE* Mitral Annular Plane Systolic Excursion, *NYHA* New York Hear Association. ^a^per cm decrease

### Multivariable Analysis and Incremental Prognostic Value

After adjustment for established clinical risk factors which were univariate predictors (age, diabetes, hypertension, NYHA class, LV mass), lateral MAPSE remained a significant independent predictor of MACE (HR = 4.384 per cm decrease; *p* = 0.020) (Table 2). Addition of lateral MAPSE to this model resulted in a significant increase in global chi-square as assessed by the likelihood ratio test (*p* = 0.03) and an integrated discrimination improvement of 0.01 (*p* = 0.01).

**Fig. 5:**
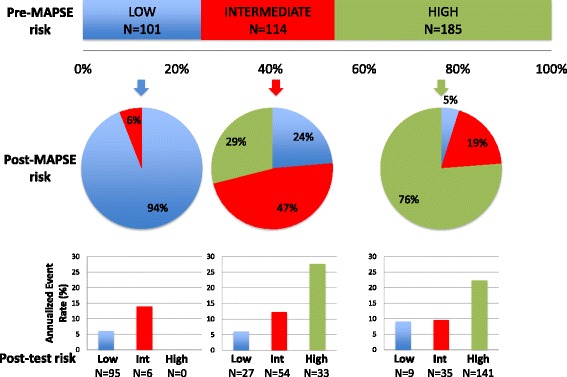
Risk reclassification. Illustration of risk reclassification by addition of lateral MAPSE to a model containing ejection fraction, age, diabetes, hypertension, NYHA class and LV mass. The upper horizontal bar graph represents the distribution of risk across categories of MACE as estimated by the model prior to adding lateral MAPSE. The pie graphs represent the proportions of patients in each pre-lateral MAPSE category reassigned to each risk category after the addition of lateral MAPSE to the risk model. The vertical bar charts at the bottom represent the annualized rates of MACE in each of the post-lateral MAPSE risk categories

Overall, the net reclassification improvement (NRI) was 0.18 (*p* = 0.006) across risk categories of MACE. As shown in Fig. 5, risk reclassification by lateral MAPSE was most effective in patients at intermediate pre-test risk, with reclassification of 24 % (27/114) of patients to low risk and 29 % (33/114) of patients to high risk, with a low (6.1 %) and high (27.8 %) annual rate of MACE, respectively. For patients at high pretest risk, lateral MAPSE reclassified 19 % (35/185) and 5 % (9/185) into moderate and low risk, with low annual rates of MACE (9.1 and 9.5 %, respectively), which were in contrast to the high annual rates of MACE (22.3 %) among patients who remained at high post-test risk.

## Discussion

In this study we have shown the feasibility of rapidly assessing MAPSE - as a surrogate of LV long axis function - during routine cine-CMR. We have also demonstrated that lateral MAPSE, is an independent predictor of major adverse cardiovascular events. To our knowledge this is the first demonstration of the prognostic value of assessing LV long axis function using universally obtained CMR images and without the need for propriety software.

### Long axis function in health and disease

Long axis function plays a fundamental role in cardiac mechanics: 1) It contributes to ventricular ejection by reducing long axis LV cavity size as the mitral annulus is pulled towards the apex [6, 7]. 2) During early diastole, the potential energy stored during systole creates ventricular suction, critical in facilitating rapid ventricular filling at low pressures in the normal heart [7, 20]. As the mitral annulus springs back to its equilibrium position it moves around the column of blood passing through the mitral valve, thus aiding ventricular filling. Through this simple mechanism, blood that was in the left atrium finds itself in the ventricle despite remaining stationary with respect to the apex and chest wall [7]. 3) The motion of the annulus towards the apex during ventricular systole has the effect of correspondingly increasing the capacity of the atrium as its floor moves downwards - thus drawing blood into the atrium from the pulmonary veins [6, 21]. 4) During atrial systole, atrial emptying and ventricular filling are further facilitated as the mitral annulus is pulled away from the apex by contraction of atrial myocardium, which is inserted into it [22].

Thus longitudinal movement of the mitral annulus is a major component of normal heart function. This has been highlighted by Carlsson et al, who used CMR to measure the contribution of longitudinal mitral plane displacement to overall stroke volume in normal subjects, elite athletes and patients with dilated cardiomyopathy [5, 6]. They suggested that although stroke volume was higher in athletes and lower in patients with cardiomyopathy, the percentage of stroke volume explained by longitudinal motion was similar at about 60 %. Longitudinal annular movement therefore appears to be a dominant contributor to left ventricular pump function in adults. Interestingly, longitudinal contribution to stroke volume in healthy children has been reported to be slightly lower at about 50 % [23]. More recently, Steding-Ehrenbor and colleagues demonstrated - using CMR - that the contribution of longitudinal motion to left ventricular stroke volume remains unchanged during moderate intensity supine exercise [24].

Possibly because of their subendocardial situation, the more longitudinal fibers are exquisitely sensitive to disturbance by various diseases and pathologies [8]. Mitral annular motion is rapidly affected by ischemia and there is a very close interaction between long axis function and coronary artery disease [4, 8]. Moreover, mitral annular plane displacement and the corresponding longitudinal contribution to stroke volume appear also to be decreased in patients with pulmonary hypertension compared to controls [25]. Indeed impairment of long axis function can be present despite preserved overall LVEF and may provide an early marker of disease. This may help explain our finding of an independent association of MAPSE with adverse cardiovascular events, independent of LVEF and other risk factors.

### MAPSE and global longitudinal strain

Since the cardiac apex is fixed with respect to the chest wall, changes in long axis function can be assessed by measuring changes in the position of the atrioventricular annulus [7]. Indeed, early studies used M-mode echocardiography to directly follow the position of the mitral annulus and measure MAPSE [26]. However, M-mode echocardiographic MAPSE assessment suffers from angle dependence issues. More recently, tissue doppler of the mitral annulus and LV strain imaging techniques have been developed to assess long axis function by echocardiography. Although clinical strain measurements initially used tissue doppler approaches, these have now been largely superseded by 2D-speckle tracking methods. Strain imaging has the potential advantage of providing segmental and global information regarding longitudinal deformation rather than the monodimensional focal data derived from MAPSE. However, these strain techniques are highly dependent on attainment of good quality imaging and there is lack of standardization between different vendors [10]. Moreover, analysis can be time consuming and requires significant operator experience [10, 27].

### Long axis function and prognosis

MAPSE measured using echocardiography has been shown to be a predictor of adverse cardiovascular outcomes in a number of conditions, including atrial fibrillation, post-myocardial infarction, heart failure, and tetralogy of Fallot [28–31]. More recently, there has been a great deal of interest in the prognostic utility of echo derived global longitudinal strain, which has been shown to predict a variety of adverse outcomes in several different populations, including acute myocardial infarction, ischemic cardiomyopathy, heart failure (with reduced or preserved ejection fraction), aortic stenosis, tetralogy of Fallot, amyloidosis, post heart transplantation, and post anthracycline therapy [10, 28, 32–38]. Echo global longitudinal strain also appears to independently predict incident atrial fibrillation in a community based cohort [39].

In this study we did not examine the relationships between MAPSE and echo derived global longitudinal strain. However, Riffel et al recently showed that displacement of the mitral annulus with respect to the cardiac apex - measured with CMR - correlates well with echo derived global longitudinal strain but did not provide outcome data [40]. We have now demonstrated for the first time that a simple, rapid measurement of lateral MAPSE from 4-chamber cine images, is independently associated with adverse outcomes in a consecutive series of patients undergoing CMR. 4-chamber cine-CMR image are obtained routinely in all CMR protocols. Moreover, the method is vendor independent and requires no special analysis software. It can be performed rapidly and shows good inter and intra observer variability.

Using CMR, Korosoglou et al have shown that strain encoded magnetic resonance imaging (SENC) provided additive prognostic information in patients undergoing dobutamine stress CMR [41]. More recently, Buss and colleagues demonstrated that LV longitudinal strain assessed with specialized CMR feature tracking software is an independent predictor of survival in dilated cardiomyopathy [42]. However, to date CMR analysis of strain has required the use of specialized software. Consequently these techniques have not achieved widespread clinical use.

### Limitations

This is a single-center, observational study and carries all of the inherent limitations of that study design. As such, it is likely that some amount of residual confounding remains, despite adjustment for a wide array of clinically relevant covariates. Patients were clinically referred for CMR at a single academic medical center and the results are not necessarily generalizable to the larger population of patients seen in the community. Our analysis used a composite of clinical end points with relatively few “hard end points”, highlighting the need for replication of these results in a larger cohort with longer follow-up. Indeed, the lack of univariable association between LGE and MACE in our population may relate to the above factors. This highlights the need for replication of these results in a larger multi-center cohort with longer follow-up allowing for analysis of hard endpoints focusing on specific populations such as those with known CAD.

In this study we prospectively chose to look only at lateral and septal MAPSE because these are the two measurements that have been recommended and historically performed in the echocardiographic literature [9]. In order to avoid multiple post-hoc statistical comparisons we chose to limit the number of variables tested to those that were prospectively selected based on the prior literature. However, given our results it would be interesting for future studies to examine the relative prognostic significance of anterior and inferior MAPSE.

In contrast to lateral MAPSE, septal MAPSE was not associated with adverse events in this study. The reason for this is not clear, although it is well appreciated from echocardiography that parameters of lateral and medial movement of the mitral annulus such as tissue doppler and MAPSE are significantly different [9, 43]. One possibility is that perhaps septal MAPSE is more affected by right ventricular interactions than lateral MAPSE and hence provides a less direct assessment of LV longitudinal function. Our MAPSE measurements did not account for possible translational movements of the mitral annulus which maybe confounding our assessment of long axis function. In the echo literature MAPSE measurements have usually been reported as absolute numbers (not indexed to body/heart size) based on simple M-mode recordings in the apical 4-chamber view. For this initial study we wanted to examine the CMR utility of this very simple measurement that has been used previously in the echo literature. However, possible refinements such as normalization to body size or measurement of apex-to-annulus length would be interesting to explore in future studies. Nevertheless our simple, rapid method for assessing long axis function was independently associated with MACE in this study. Plasma BNP levels were not measured at the time of the scanning and hence could not be included in our predictive models. Future studies need to replicate these results in a larger cohort with longer follow-up and looking at “hard endpoints” before routine clinical measurement of lateral MAPSE by CMR can be advocated.

## Conclusions

Reduced long axis function assessed with lateral MAPSE during routine cine-CMR is an independent predictor of MACE.

## Abbreviations

CMR, cardiovascular magnetic resonance; EF, ejection fraction; LGE, late gadolinium enhancement; LV, left ventricle; MACE, major adverse cardiac events; MAPSE, mitral annular plane systolic excursion; NRI, net reclassification improvement; NYHA, New York heart association
